# Multi-Targeted Intervention of *Eucommia ulmoides* and Its Bioactive Constituents Against Metabolic Syndrome: From Molecular Mechanisms and Gut Microbiota Modulation to Clinical Translation

**DOI:** 10.3390/metabo16060411

**Published:** 2026-06-12

**Authors:** Fanjia Cheng, Chenghao Lv, Yuhang Yi, Dongsheng Wang, Wenbo Wang, Tao Li, Runze Zhou, Qili Li, Si Qin

**Affiliations:** 1 Laboratory of Food Function and Nutrigenomics, College of Food Science and Technology, Hunan Agricultural University, Changsha 410128, China; chengfanjia@stu.hunau.edu.cn; 2 Hunan Provincial Key Laboratory of Liver Visceral Manifestation in Traditional Chinese Medicine, Institute of Integrative Medicine, Department of Integrated Traditional Chinese and Western Medicine, Xiangya Hospital, Central South University, Changsha 410008, China; lvchenghao@xiangya.com.cn (C.L.); wdsh66@aliyun.com (D.W.); wangwenbo@csu.edu.cn (W.W.); 3 College of Bioscience and Biotechnology, Hunan Agricultural University, Changsha 410128, China; yiyuhang@stu.hunau.edu.cn (Y.Y.); zhourunze@stu.hunau.edu.cn (R.Z.); 4 Hunan Institute of Agricultural Product Processing and Quality Safety, Hunan Academy of Agricultural Sciences, Changsha 410125, China; litao@hunaas.cn

**Keywords:** *Eucommia ulmoides*, metabolic syndrome, chlorogenic acid, lignans, insulin resistance, phytochemicals

## Abstract

**Background/Objectives:** Metabolic syndrome (MetS) is a pressing global health challenge comprising obesity, hyperglycemia, hypertension, and hyperlipidemia. Conventional polypharmacy often presents long-term compliance issues and side effects. *Eucommia ulmoides* Oliv., a traditional medicinal and edible plant rich in iridoids, lignans, flavonoids, and polysaccharides, has emerged as a promising natural intervention. This review aims to systematically summarize the bioavailability and multifaceted pharmacological mechanisms of *E. ulmoides* and its bioactive components in alleviating MetS. **Methods:** We comprehensively reviewed the recent in vitro and in vivo literature to map the functional evidence, specific signaling pathways, and gut microbiota–host interactions associated with *E. ulmoides* extracts and its key phytochemicals (e.g., asperuloside) against various metabolic dysfunctions. **Results:** Current evidence indicates that *E. ulmoides* operates through a “multi-component, multi-target, and multi-pathway” paradigm. For hyperlipidemia and obesity, it activates hepatic lipid metabolism (PPARα/CPT1A, FXR/CYP7A1) and mitigates oxidative stress (Nrf2/ARE). Furthermore, it dose-dependently reshapes the gut microbiota by enriching beneficial bacteria like Akkermansia and increasing butyrate production, exerting profound gut–liver axis regulation. It also ameliorates hypertension by activating the ACE2-Ang-(1–7)-Mas axis, improves insulin resistance via the AMPK/PI3K/Akt cascade, and manages hyperuricemia by modulating XOD and renal transporters. Notably, the low oral bioavailability of its glycosides highlights the crucial role of gut microbial hydrolysis in its efficacy. **Conclusions**: *E. ulmoides* holds substantial therapeutic potential as a multi-target natural supplement for MetS. However, future translational applications necessitate large-scale randomized clinical trials, multi-omics studies to further clarify host–microbiome interactions, and the development of standardized formulations to ensure clinical efficacy.

## 1. Introduction

Metabolic syndrome (MetS) represents a complex, multifactorial cluster of metabolic derangements characterized by the cardinal features of central adiposity, dysglycemia, elevated blood pressure, and atherogenic dyslipidemia—encompassing hypertriglyceridemia and/or reduced high-density lipoprotein cholesterol (HDL-C) [1]. The clinical significance of metabolic syndrome lies in its convergence of features shared by multiple chronic disorders. It is a major risk factor for a range of diseases, including cardiovascular disease, type 2 diabetes mellitus, and non-alcoholic fatty liver disease, markedly increasing both mortality and overall disease burden [2,3]. The global prevalence of metabolic syndrome and associated disorders has become increasingly alarming. A 2022 modeling study estimated that in 2020, metabolic syndrome affected approximately 2.8% of children and 4.8% of adolescents worldwide [4,5]. Type 2 diabetes mellitus, a major consequence of metabolic syndrome, continues to increase in prevalence globally and is projected to affect more than 600 million people by 2030, thereby imposing a profound burden on healthcare systems worldwide [2]. At present, the clinical management of metabolic syndrome is largely centered on lifestyle interventions, including dietary regulation and enhanced physical activity [6]. In addition, the management of metabolic syndrome often involves pharmacotherapy directed at specific components, including antihypertensive, antidiabetic, and lipid-lowering medications [2]. Nevertheless, these approaches are constrained by significant drawbacks. Lifestyle interventions typically exhibit poor long-term adherence, whereas combination pharmacotherapy (polypharmacy) not only increases the risk of adverse drug interactions but also rarely addresses the underlying pathological processes driving metabolic dysregulation [2,3].

Consequently, the development of novel therapeutic strategies that are multi-targeted, safe, and capable of simultaneously correcting multiple metabolic abnormalities has become a major focus of research. Against this backdrop, natural products derived from plants and foods, along with their bioactive components, are attracting increasing attention due to their immense therapeutic potential. Extensive evidence suggests that many natural compounds can alleviate various manifestations of metabolic syndrome through multiple mechanisms, including anti-inflammatory and antioxidant effects, modulation of the gut microbiota, and improvement of insulin sensitivity [7]. These advances underscore the importance of systematically investigating the role of traditional medicinal plants, such as European elm, in the prevention and regulation of metabolic syndrome.

*Eucommia ulmoides* Oliv. is a monotypic species endemic to China, distributed primarily in provinces south of the Qinling Mountains, including Guizhou, Sichuan, Hunan, Shaanxi, Hubei, and Jiangxi, across an altitudinal range of 50–2500 m [8]. At present, its annual production in China is approximately 1408.4 tons, with Guizhou, Sichuan, Hunan, and Jiangxi serving as the principal regions for standardized cultivation [9]. In terms of safety, systematic toxicological evaluations have demonstrated that *E. ulmoides* extracts exhibit no mutagenicity in the Ames test, bone marrow micronucleus test, or chromosomal aberration assay, with a maximum tolerated dose of no less than 168 g/kg, equivalent to approximately 1260 times the adult clinical dose. Long-term administration at high doses (56 g/kg) may induce nephrotoxicity, whereas damage caused by lower doses (11.2 g/kg) was reversible within 5 weeks after drug withdrawal [10]. In clinical studies, a standardized *E. ulmoides* bark extract containing 8% pinoresinol diglucoside at a daily dose of 1 g for 2 weeks reduced blood pressure by 7.5/3.9 mmHg and was well tolerated [11]. Pharmacokinetic studies further showed that the iridoid constituents geniposidic acid and aucubin are rapidly absorbed after oral administration, with half-lives of 2.37 ± 1.72 h and 2.27 ± 1.14 h, respectively [12]. Nevertheless, the major bioactive constituents of *E. ulmoides* are predominantly present in glycosidic forms, resulting in low oral bioavailability and requiring hydrolysis by the gut microbiota into aglycones prior to absorption [13]. UPLC–Q-TOF–MS analysis identified 20 prototype metabolites in plasma, among which 6 compounds displayed rapid absorption, high systemic exposure, and fast elimination profiles [14].

Within the theoretical framework of traditional Chinese medicine, *E. ulmoides* has long occupied an important position and has been regarded by successive generations of physicians as a medicinal herb that nourishes the liver and kidneys and strengthens muscles and bones. Its medicinal use can be traced back to the Shennong Bencao Jing, the earliest extant classic of Chinese materia medica [15]. However, providing a modern scientific interpretation of its traditional functions has remained a central challenge in the modernization of traditional Chinese medicine. With advances in analytical technologies and increasingly sophisticated research approaches, the bioactive constituents of *E. ulmoides* and their molecular mechanisms have been progressively elucidated, thereby providing a scientific basis for its application in the prevention and treatment of modern chronic diseases such as metabolic syndrome. Available studies indicate that the entire plant—including the bark, leaves, male flowers, and seeds—constitutes a rich natural reservoir of bioactive compounds, including lignans, iridoids, phenylpropanoids, flavonoids, and polysaccharides. These constituents confer diverse pharmacological activities on *E. ulmoides*, among which anti-inflammatory and antioxidant effects are considered common biological foundations underlying many of its therapeutic properties and have become a major focus of recent research.

Accordingly, this review aims to provide a comprehensive and up-to-date overview of the therapeutic potential of *Eucommia ulmoides* in metabolic syndrome. We systematically summarize its principal bioactive constituents, analyze the underlying molecular mechanisms targeting the key components of metabolic syndrome (hyperlipidemia, hyperuricemia, hyperglycemia, and hypertension), and discuss the emerging role of gut microbiota modulation in its therapeutic effects. Furthermore, we highlight current research limitations and propose future perspectives to facilitate the clinical translation of *E. ulmoides* from a traditional medicinal plant to an evidence-based natural intervention for metabolic syndrome.

Relevant studies were identified from the published literature and were qualitatively synthesized to provide an updated overview of the pharmacological properties and therapeutic applications of *Eucommia ulmoides*.

## 2. Pathophysiological Mechanisms of Metabolic Syndrome

Metabolic syndrome (MetS) is a complex disorder characterized by the clustering of multiple metabolic abnormalities, with insulin resistance at its pathological core. Its development reflects a multifactorial interplay involving genetic susceptibility, chronic low-grade inflammation, oxidative stress, and dysregulation of the gut microbiota [16]. Here, we focus on the four hallmark features of MetS, emphasizing the central role of insulin resistance in orchestrating the syndrome’s pathophysiological landscape.

### 2.1. The Pathophysiological Mechanisms of Hyperglycemia

**Figure 1:**
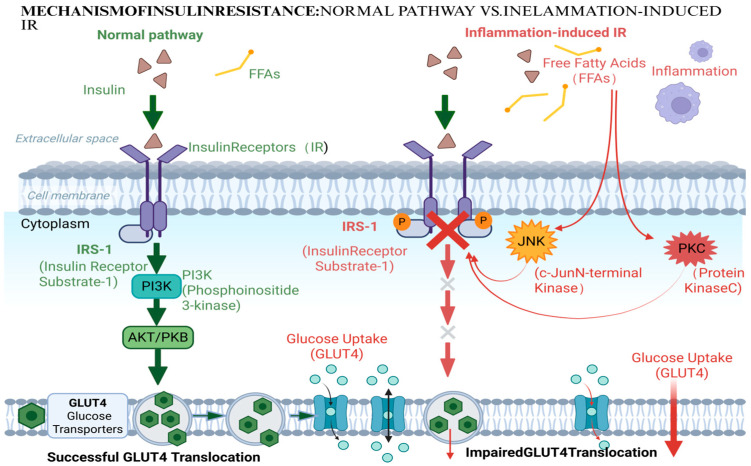
Diagram of the Pathological Mechanisms of Hyperglycemia.

Insulin resistance (IR) is the pathophysiological hallmark of MetS and is defined by reduced sensitivity of target tissues to insulin-mediated biological effects [17]. Under normal conditions, insulin binding to its receptor triggers the IRS-1/PI3K/Akt pathway, leading to GLUT4 translocation and subsequent glucose uptake [18]. In obesity, adipose tissue-derived free fatty acids (FFAs) and pro-inflammatory cytokines, such as TNF-α and IL-6, activate JNK and PKC, thereby promoting serine phosphorylation of IRS-1 and disrupting the insulin signaling cascade. This impairment contributes to defective glucose disposal in skeletal muscle and uncontrolled gluconeogenesis in the liver [19]. The principal mechanisms involved are summarized in Figure 1.

### 2.2. The Pathophysiological Mechanisms of Hyperlipidemia

**Figure 2:**
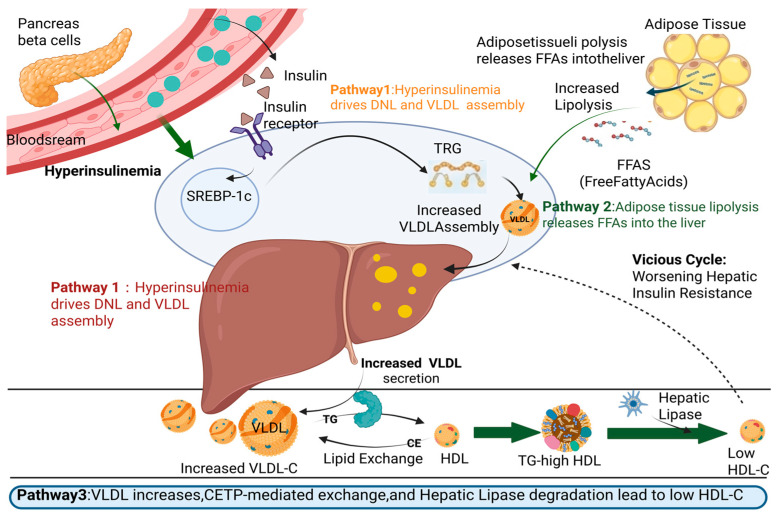
Diagram of the Pathological Mechanisms of Hyperlipidemia.

Hepatic insulin resistance serves as a central driver of dyslipidemia. Hyperinsulinemia activates SREBP-1c, thereby promoting de novo lipogenesis in the liver and enhancing VLDL assembly and secretion. Elevated plasma triglycerides subsequently induce CETP-mediated lipid exchange, resulting in triglyceride-enriched HDL that is rapidly catabolized by hepatic lipase, leading to reduced HDL-C levels [20]. Concurrently, enhanced lipolysis in adipose tissue continuously delivers free fatty acids (FFAs) to the liver, further stimulating hepatic lipogenesis and VLDL overproduction. This establishes a vicious cycle that underlies hypertriglyceridemia and hepatic steatosis [21]. The pathological mechanisms are illustrated in Figure 2.

### 2.3. The Pathophysiological Mechanisms of Hypertension

**Figure 3:**
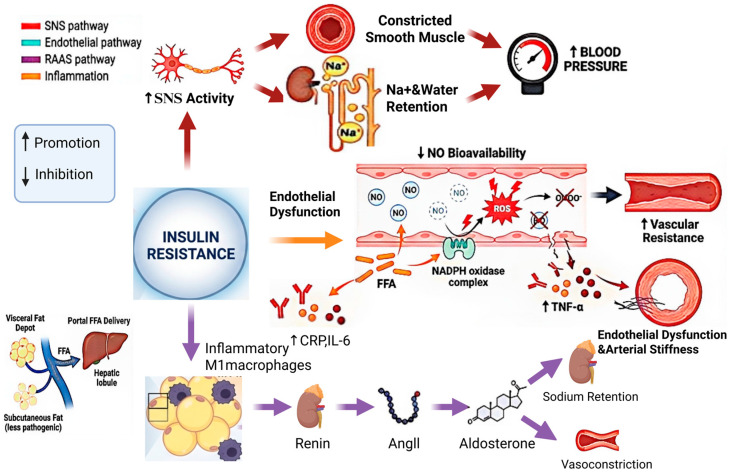
Diagram of the Pathophysiological Mechanisms of Hypertension.

Insulin resistance promotes the development of hypertension through multiple interrelated mechanisms. Mechanistically, this process involves free fatty acid (FFA)-induced generation of reactive oxygen species (ROS), which scavenge NO, as well as the loss of insulin-mediated vasodilatory signaling [21]. In addition, obesity—particularly visceral adiposity—contributes to hypertension by activating the renin–angiotensin–aldosterone system (RAAS), thereby further promoting sodium retention and vasoconstriction. Chronic low-grade inflammation also participates in the pathogenesis of hypertension, as elevated inflammatory markers such as C-reactive protein (CRP) and IL-6 exacerbate endothelial dysfunction and increase arterial stiffness [22]. Importantly, because visceral adipose tissue releases FFAs directly into the portal circulation, it exerts a more pronounced impact on insulin resistance and hypertension than subcutaneous fat [21]. The pathological mechanisms are illustrated in Figure 3.

### 2.4. The Pathophysiological Mechanisms of Hyperuricemia

**Figure 4:**
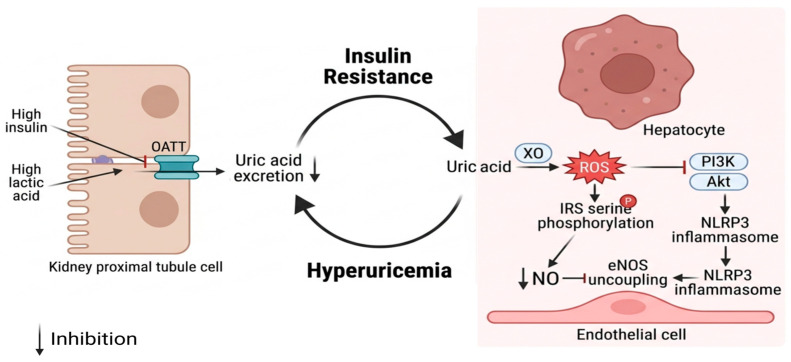
Diagram of the Pathological Mechanisms of Hyperuricemia.

Insulin resistance (IR) and hyperuricemia are closely interrelated in a bidirectional manner. On one hand, compensatory hyperinsulinemia reduces renal uric acid excretion, establishing a deleterious feedback loop [23]. Mechanistically, this involves insulin-mediated regulation of organic anion transporters in the renal proximal tubules, as well as competitive inhibition of uric acid excretion by lactate accumulation [23]. On the other hand, hyperuricemia can exacerbate insulin resistance via oxidative stress pathways. During uric acid production, xanthine oxidase (XO) generates reactive oxygen species (ROS) that preferentially induce serine phosphorylation of IRS proteins rather than tyrosine phosphorylation, thereby impairing their interaction with insulin receptors and downstream PI3K/Akt signaling. Furthermore, hyperuricemia diminishes endothelial nitric oxide (NO) availability and promotes eNOS uncoupling, leading to “vascular insulin resistance” [24]. Recent studies also indicate that hyperuricemia modulates hepatic steatosis and insulin resistance through NLRP3 inflammasome-dependent mechanisms [25], and Mendelian randomization analyses have confirmed a causal link between insulin resistance and elevated uric acid levels [26]. The pathological mechanisms are illustrated in Figure 4.

## 3. Principal Bioactive Constituents and Chemical Structures of *Eucommia ulmoides*

Widely distributed throughout China and characterized by high yield, *Eucommia ulmoides* has long been valued as a traditional medicinal resource [27]. Its medicinal use dates back thousands of years and is documented in numerous classical medical texts, with its therapeutic relevance reinforced by extensive clinical experience accumulated over time. More recently, its medicinal value has been further validated by modern pharmacological research and evidence-based clinical studies, leading to its standardized and widespread application in contemporary clinical practice [28]. Phytochemically, *E. ulmoides* is rich in structurally diverse bioactive constituents, primarily including lignans, iridoids, polyphenols, flavonoids, and polysaccharides. These compounds have been shown to underlie a wide range of pharmacological activities, such as antihypertensive effects, improvement of glucose metabolism, anti-inflammatory and analgesic actions, hepato protection, and suppression of tumor cell proliferation [28,29,30]. The Key Active Ingredients in Eucommia in Table 1.

### 3.1. Iridoid

Another class of key bioactive constituents in *Eucommia ulmoides*, commonly referred to as “Eucommia gum,” is predominantly enriched in the leaves and young shoots. Chemically, these compounds belong to monoterpenoid derivatives, featuring a fused cyclopentane[c]pyran ring system as their core scaffold [31]. Similar to lignans, iridoids in the plant mainly exist as glycosides, known as iridoid glycosides, which confer enhanced water solubility. The presence of hemiacetal or hemiketal moieties within their structures renders these compounds chemically labile, making them susceptible to hydrolysis or rearrangement under acidic or enzymatic conditions [15].

Iridoid research in *E. ulmoides* has a long history, with continuous identification of new structures, reflecting the chemical diversity of this class. Among these, three iridoids are considered emblematic: geniposidic acid, aucubin, and geniposide [15,31]. Geniposidic acid is the most abundant iridoid in *E. ulmoides* and serves as a biosynthetic precursor for many other complex iridoids. Its hallmark feature is a carboxyl group attached to the cyclopentane ring. Aucubin shares a similar structural framework with geniposidic acid, but contains an additional hydroxyl group on the cyclopentane ring and differs in the position of its enol ether double bond; it is also widely distributed in other plant species. Geniposide represents the methyl ester derivative of geniposidic acid, in which the carboxyl group is methylated. The structures of these core iridoids have been rigorously confirmed through comprehensive spectroscopic analyses [32]. Beyond these principal compounds, numerous other monomeric iridoid glycosides have been isolated from *E. ulmoides*, including ajugoside, reptoside, harpagide acetate, and a novel compound named eucommiol, characterized as a unique cyclopentanetetrol [15,31]. Variations in the type, number, and position of substituents across these molecules underpin the remarkable structural diversity of iridoids in *E. ulmoides*.

Pharmacokinetically, geniposidic acid and aucubin are rapidly absorbed yet poorly bioavailable. In rats, geniposidic acid reaches Cmax (895–1580 ng/mL) at 1.17–1.33 h with T1/2 of 0.82–0.89 h, while aucubin peaks earlier (Tmax 0.42 h, T1/2 0.34–0.38 h) [14]. Their hydrophilic nature (log P ≈ −2.09 and −2.25) limits membrane permeability [33]. Structurally related geniposide has an absolute oral bioavailability of merely ~9.67% due to extensive first-pass metabolism [34]. Consequently, gut microbial deglycosylation to aglycones (e.g., genipin) constitutes a critical activation step that substantially influences systemic exposure [34,35].

### 3.2. Phenolic Acids and Flavonoids

In addition to lignans, *Eucommia ulmoides* is rich in a diverse array of simple phenolics and polyphenolic compounds, among which flavonoids represent a particularly important family. These constituents are widely distributed in the leaves, bark, and flowers of *E. ulmoides* and contribute substantially to its antioxidant, antihypertensive, and lipid-lowering activities [36].

Phenolic acids: Phenolic acids constitute one of the simplest yet biologically active classes of phenolic compounds in *E. ulmoides*. Based on their core structures, they can be broadly categorized into benzoic acid derivatives (C6–C1) and cinnamic acid derivatives (C6–C3). Chlorogenic acid is a highly abundant and representative phenolic acid in *E. ulmoides* leaves [36,37,38]. Structurally, it is formed by esterification of caffeic acid with quinic acid. Both chlorogenic acid and its hydrolysis product, caffeic acid, exhibit potent antioxidant activity. In addition to chlorogenic acid, several other phenolic acids have been identified in *E. ulmoides*, including caffeic acid, ferulic acid, p-coumaric acid, gallic acid, and vanillin [37]. Although structurally simple, these compounds possess phenolic hydroxyl groups that confer pronounced free-radical-scavenging and metal-chelating properties.

Flavonoids: Flavonoids share a common C6–C3–C6 carbon framework and can be further classified into multiple subclasses, including flavonols, flavones, flavanones, and isoflavones, according to the oxidation state of the central C ring, the pattern of inter-ring linkage, and the nature of their substituents [39,40,41]. Flavonoids are also abundant in *E. ulmoides*. Flavonols represent the predominant flavonoid subclass in this species, with quercetin and kaempferol being the most representative compounds [42,43,44]. These molecules frequently occur in glycosylated forms, such as rutin (quercetin-3-O-rutinoside), astragalin (kaempferol-3-O-glucoside), isoquercitrin (quercetin-3-O-glucoside), and quercetin-3-O-β-D-galactoside [32,39,42]. Glycosylation not only influences solubility and absorption but may also be critical for bioactivity. In addition, other flavonoids, including luteolin and its glycosides, hesperetin, naringenin, and catechin, have also been identified in *E. ulmoides* [37,40,45].

The structures of these phenolic and flavonoid compounds are typically characterized by ultraviolet (UV) spectroscopy, infrared (IR) spectroscopy, nuclear magnetic resonance (NMR), and mass spectrometry (MS) [46,47]. A common structural hallmark of these metabolites is the presence of multiple phenolic hydroxyl groups, which constitute the principal functional moieties underlying their antioxidant and related biological activities.

Regarding oral bioavailability, chlorogenic acid shows biphasic absorption: human ileostomy studies demonstrate that approximately one-third (33 ± 17%) is absorbed in the small intestine, while the majority reaches the colon for microbial hydrolysis to caffeic and quinic acids, followed by conversion to hippuric acid (~57% urinary recovery) [48,49,50]. Flavonoid glycosides such as rutin and isoquercitrin similarly require microbial or enzymatic deglycosylation prior to absorption; quercetin aglycone has ~53% intestinal absorption in rats but undergoes extensive hepatic first-pass metabolism (sulfation, glucuronidation, and methylation), significantly limiting systemic bioavailability [51].

### 3.3. Lignan

Lignans are among the hallmark and most abundant bioactive constituents of *Eucommia ulmoides*, particularly in the bark [36,52]. They are a class of natural phenolic compounds biosynthetically derived from the dimerization of two phenylpropanoid (C6–C3) units, typically linked through the β-carbon atoms of their side chains (8–8′ linkage). Depending on the mode of coupling and the extent of cyclization, lignans can be further classified into several structural subtypes [52]. In *E. ulmoides*, these compounds occur predominantly in glycosylated forms, which not only influence their solubility and bioavailability but may also modulate their biological activities [53].

According to differences in their carbon skeletons, the lignans identified in *E. ulmoides* can be broadly divided into several major categories. Furofuran lignans represent the most common subclass and are characterized by a bicyclic system composed of two fused tetrahydrofuran rings [36]. Tetrahydrofuran lignans, by contrast, possess a single tetrahydrofuran ring as the core scaffold. A representative compound in this group is (−)-olivil and its glycosides, such as olivil 4′,4″-di-O-β-D-glucopyranoside [54]. Structurally, olivil differs substantially from furofuran lignans such as pinoresinol, as its two phenylpropanoid units are linked in a distinct manner to generate a tetrahydrofuran ring system.

In addition to these major subclasses, several structurally unusual lignans have been identified from *E. ulmoides* as advances in isolation and structural elucidation techniques have accelerated the discovery of novel metabolites. For example, cycloolivil, which contains an oxirane bridge, and its epimer (+)-epicycloolivil have been isolated from the bark of *E. ulmoides* [53,55]. A novel lignan designated Eucomin A was subsequently identified as (+)-medioresinol 4′-O-β-D-glucopyranoside [54], and the structurally distinctive compound Noreucol A has also been reported [53]. The discovery of these previously uncharacterized lignans has greatly expanded the known chemical diversity of lignans in *E. ulmoides* and provides an important material basis for investigating their additional biological activities.

Pharmacokinetic studies reveal that pinoresinol diglucoside (PDG) displays poor oral bioavailability in its intact glycosylated form (Cmax 45.3 ng/mL at 0.5 h, T1/2 1.15 h in rats), whereas its aglycone pinoresinol achieves higher exposure (Cmax 113.1 ng/mL, T1/2 3.57 h) [56]. This disparity underscores the pivotal role of gut microbial deglycosylation in lignan bioactivation. Following ingestion, PDG undergoes sequential microbial biotransformation: deglycosylation to pinoresinol, reduction to lariciresinol, and ultimately conversion to the mammalian lignans enterodiol and enterolactone [57,58]. The bioavailability of these enterolignans is highly variable among individuals (estimated at 20–40%), reflecting inter-individual differences in gut microbiota composition [59].

### 3.4. Polysaccharide

Polysaccharides represent another class of bioactive macromolecules in *Eucommia ulmoides*, particularly abundant in its leaves and bark [36]. Unlike small-molecule compounds, the bioactivity of polysaccharides is not solely determined by their chemical composition; it is also intricately influenced by molecular weight, glycosidic architecture—including the connectivity and branching of the main and side chains—and higher-order spatial conformation [60,61]. Studies on *E. ulmoides* polysaccharides have highlighted their distinctive roles in immunomodulation, antioxidation, and regulation of gut microbiota.

*E. ulmoides* polysaccharides are structurally complex and heterogeneous, and investigations into their fine structures are ongoing. Monosaccharide composition analysis of hydrolyzed polysaccharides has revealed a diverse profile, predominantly comprising glucose (Glc), galactose (Gal), mannose (Man), arabinose (Ara), and rhamnose (Rha). Minor components often include xylose (Xyl), fucose (Fuc), and uronic acids such as galacturonic acid (GalA) and glucuronic acid (GluA) [62,63]. Notably, the monosaccharide composition and molar ratios can vary significantly depending on the plant tissue (leaf vs. bark), harvest time, and extraction/purification methodologies [61]. Structural characterization using methylation analysis and nuclear magnetic resonance (NMR) spectroscopy has elucidated the complex glycosidic linkages of *E. ulmoides* polysaccharides. The main chains are suggested to consist of (→4)-β-D-Glcp-(1→, →4)-β-D-Manp-(1→, or →4)-α-D-GalpA-(1→) motifs, which are further substituted at various positions (e.g., O-2, O-3, O-6) with side chains composed of arabinose, galactose, and xylose [61]. This highly branched architecture establishes intricate three-dimensional networks that provide the spatial framework necessary for interactions with cell-surface receptors, such as Toll-like receptors, thereby underpinning their immunomodulatory activities. Acidic polysaccharide fractions containing uronic acids have been particularly associated with enhanced biological activity [62]. The superior bioactivity of acidic polysaccharide fractions stems from the distinctive chemical properties of uronic acid residues (GalA and GluA). The carboxyl group (-COOH) at the C-6 position imparts a negative charge under physiological pH, conferring three critical functional advantages over neutral polysaccharides [64]. First, carboxyl groups serve as potent metal ion chelators (Fe^2+^, Cu^2+^), suppressing Fenton-type ROS generation at the source and enhancing antioxidant capacity—an effect that increases proportionally with uronic acid content [65,66]. The anomeric hydrogen atoms can also directly quench free radicals [66]. Second, the negative surface charge facilitates electrostatic interactions with immune cell receptors (TLR4, Dectin-1), triggering MAPK and PI3K/Akt signaling cascades that enhance phagocytosis, cytokine secretion (TNF-α, IL-6, IL-1β, IFN-β), and NO production [67]. The GalA-rich HG and RG-I domains represent the primary immunomodulatory structural motifs [67]. Third, uronic acids increase hydrophilicity and water-holding capacity, improving solubility and enabling three-dimensional network formation for optimal receptor engagement [61,65]. Notably, low-molecular-weight oligogalacturonides exhibit superior radical-scavenging capacity, indicating that both content and accessibility of uronic acid residues determine activity [65].

Unlike small-molecule constituents, *E. ulmoides* polysaccharides are not absorbed intact in the upper gastrointestinal tract due to their high molecular weight and hydrophilic nature. Instead, they serve as fermentable substrates for colonic microbiota, producing short-chain fatty acids (SCFAs) such as butyrate, propionate, and acetate [61,68]. These microbial metabolites are rapidly absorbed in the hindgut and contribute substantially to host energy metabolism and immune regulation [69]. Thus, the bioactivity of *E. ulmoides* polysaccharides is indirect and microbiota-dependent, contrasting with the direct absorption pathways of iridoids and flavonoids.

### 3.5. Others

In addition to the extensively studied classes of active constituents in *Eucommia ulmoides*—namely iridoids, polyphenols and flavonoids, lignans, and polysaccharides—the plant also contains a variety of other bioactive compounds. Although these constituents have been less extensively quantified or investigated compared with the aforementioned groups, they remain integral to the overall chemical profile of *E. ulmoides* and contribute synergistically to its pharmacological effects. These include steroids, triterpenoids, and amino acids.

Among the steroidal compounds, plant sterols are predominant, with representative examples including β-sitosterol, campesterol, and stigmasterol. These molecules share a characteristic cyclopentanoperhydrophenanthrene skeleton, typically hydroxylated at the C-3 position. β-Sitosterol is the most abundant phytosterol in *E. ulmoides*, structurally analogous to cholesterol, differing only by an ethyl substitution at C-24 [70].

Numerous triterpenoids and their saponins have also been isolated from *E. ulmoides*, primarily belonging to the ursane and oleanane pentacyclic triterpenoid families. Representative compounds include oleanolic acid, ursolic acid, and the ursane-type triterpenoid ulmoidol. Oleanolic acid is characterized by a pentacyclic triterpenoid scaffold bearing a carboxyl group at C-28, whereas ursolic acid shares a similar pentacyclic framework but differs in the methyl configurations at C-19 and C-20 [71]. Functional studies have demonstrated that oleanolic acid can activate the PPARα pathway, thereby promoting fatty acid oxidation and suppressing lipogenesis, ultimately ameliorating non-alcoholic fatty liver disease; it also enhances insulin sensitivity and improves glucose tolerance [72]. Ursolic acid has been shown to inhibit adipocyte differentiation and attenuate obesity, potentially via suppression of NF-κB signaling and reduction in pro-inflammatory cytokine secretion [73].

Accordingly, this review focuses primarily on four major classes of constituents in *Eucommia ulmoides*, iridoids, lignans, phenolics and flavonoids, and polysaccharides. These classes are structurally distinct yet mechanistically complementary, collectively underpinning the characteristic pharmacological paradigm of *E. ulmoides* as a multi-component, multi-target, and multi-pathway medicinal plant. Together, they not only help explain the broad range of therapeutic effects historically attributed to *E. ulmoides* in traditional medicine, but also highlight its considerable potential in modern medicine for the prevention and treatment of metabolic syndrome, neurodegenerative disorders, and immune-related diseases. Future research should place greater emphasis on elucidating the structure–activity relationships, in vivo metabolic fates, precise molecular targets, and synergistic or antagonistic interactions among these constituents, thereby providing a more robust scientific foundation for the precision-medicine application of *E. ulmoides* and the development of high-value functional products.

The oral bioavailability of these minor constituents remains less characterized compared to the major classes. Phytosterols such as β-sitosterol are absorbed at low levels (~5% in humans) due to their structural similarity to cholesterol and competition with endogenous sterols for intestinal transporters. Triterpenoids including oleanolic acid and ursolic acid generally exhibit poor aqueous solubility and low membrane permeability, resulting in limited oral absorption; formulation strategies such as lipid-based delivery systems or nanoparticle encapsulation are often required to enhance their bioavailability [72,74].

#### 3.5.1. Pharmacokinetics of Bioactive Aglycones

Table 2 summarizes key pharmacokinetic parameters of *E. ulmoides* constituents and their aglycone metabolites in rodent models.

Aglycones exhibit distinct pharmacokinetic profiles from parent glycosides. Following oral administration of geniposide, the aglycone genipin shows lower Cmax and AUC but comparable Tmax, suggesting rapid microbial hydrolysis followed by extensive hepatic first-pass metabolism [75]. Pinoresinol displays higher systemic exposure than PDG (AUC0–∞ 332.4 vs. 121.1 ng·h/mL) with prolonged elimination (T1/2 3.57 vs. 1.15 h), consistent with improved lipophilicity after deglycosylation [56]. Aucubin oral bioavailability is moderate (~19.3%) but limited by rapid degradation in gastric acid (t1/2 5.1–14.8 h at pH 1.2–2.0) and low lipophilicity [76].

#### 3.5.2. Modulating Factors

Food matrix. Co-ingestion with food alters polyphenol absorption kinetics. High-fat meals reduce chlorogenic acid bioavailability, while carbohydrate-rich meals modify release kinetics without affecting total absorption [77]. Milk proteins bind polyphenols, decreasing urinary recovery [34]. For *E. ulmoides* glycosides dependent on colonic hydrolysis, food matrix effects may shift the site of microbial biotransformation.

Gastric pH. Aucubin undergoes rapid acid-catalyzed degradation at pH 1.2–2.0 [76]. Inter-individual variation in gastric pH (age, PPI use, *H. pylori* infection) may alter glycoside survival to the colon, representing an underexplored source of variability.

Individual microbiota. Gut microbiota composition is the dominant driver of inter-individual variability in polyphenol metabolism [78]. For lignans, conversion to enterolignans (enterodiol, enterolactone) requires specific bacterial taxa [58]. In humans, enterodiol and enterolactone appear in plasma 8–10 h after SDG ingestion, with Tmax of 14.8 h and 19.7 h, and elimination half-lives of 4.4 h and 12.6 h, respectively [79]. Only 20–40% of individuals are efficient enterolignan producers [59]. Stratification by microbiome profiles has been proposed to reduce variability in clinical trials [78].

#### 3.5.3. Gaps in In Vivo Metabolism

Critical gaps remain: (i) bacterial species and enzymatic pathways for *E. ulmoides*-specific iridoid and lignan deglycosylation are uncharacterized; (ii) hepatic UGT/SULT isoforms involved in aglycone conjugation and their genetic polymorphisms are unidentified; (iii) enterohepatic recycling contribution to overall bioavailability is unknown; (iv) disease effects on *E. ulmoides* pharmacokinetics are unexplored; and (v) no human pharmacokinetic studies of *E. ulmoides* aglycones exist.

### 3.6. Synergistic and Antagonistic Interactions Among Bioactive Constituents

The therapeutic efficacy of *Eucommia ulmoides* has traditionally been attributed to its major bioactive constituents, including iridoids, lignans, flavonoids, and polysaccharides. However, an emerging body of evidence suggests that the overall pharmacological activity of *E. ulmoides* extracts frequently exceeds the sum of their isolated components, underscoring the importance of multi-component interactions [80,81]. This phenomenon aligns with the broader paradigm in phytomedicine wherein whole-plant preparations often outperform single purified compounds due to synergistic, additive, or—less frequently—antagonistic interactions among co-occurring metabolites [82].

Pharmacodynamic synergy among *E. ulmoides* constituents may arise through multi-target modulation of disease pathways. For instance, iridoids (e.g., aucubin, geniposidic acid) and lignans (e.g., pinoresinol diglucoside, PDG) have each been independently shown to exert anti-inflammatory effects via distinct molecular targets: aucubin suppresses NF-κB p65 nuclear translocation and IκBα degradation [83], whereas PDG inhibits the AKT/mTOR/NF-κB axis to attenuate cardiac hypertrophy [84]. When present together in the crude extract, these compounds may act on complementary nodes of the same signaling network, producing enhanced anti-inflammatory and anti-fibrotic outcomes that neither achieves alone at equivalent doses. Similarly, flavonoids such as quercetin and chlorogenic acid contribute antioxidant capacity through free-radical scavenging and metal chelation, while iridoids activate the Nrf2/ARE pathway to upregulate endogenous antioxidant enzymes [85,86]. The concurrent presence of these structurally distinct classes may thus provide redundant yet complementary cytoprotection against oxidative stress in metabolic syndrome.

Pharmacokinetic synergy represents another critical dimension. The oral bioavailability of iridoid glycosides and lignan glycosides in *E. ulmoides* is notably low owing to their hydrophilic sugar moieties [13]. Polysaccharides and certain flavonoid glycosides present in the whole extract may function as natural solubilizers or absorption enhancers, improving the dissolution and intestinal permeability of these active aglycones after microbial hydrolysis [87]. In addition, polyphenols can inhibit intestinal efflux transporters (e.g., P-glycoproteins), thereby increasing the systemic exposure of co-administered bioactive compounds. This “carrier” or “facilitator” role of seemingly non-active matrix components exemplifies how the chemical complexity of *E. ulmoides* extracts can enhance rather than dilute therapeutic potency.

Gut microbiota-mediated synergy is particularly relevant to *E. ulmoides*. Polysaccharides, which are poorly absorbed in the upper gastrointestinal tract, serve as prebiotics that selectively enrich beneficial bacteria such as Akkermansia, Lactobacillus, and butyrate-producing Ruminococcaceae [68,86]. These microbial shifts not only improve intestinal barrier integrity but also enhance the biotransformation of iridoid and lignan glycosides into their pharmacologically active aglycone forms (e.g., genipin from geniposide) [13]. Consequently, the presence of polysaccharides creates a favorable gut environment that amplifies the bioactivity of other co-ingested *E. ulmoides* constituents—a form of indirect synergy mediated by host–microbiome co-metabolism.

Antagonistic interactions, though less emphasized, have also been documented in phytochemical mixtures and warrant consideration. In the context of *E. ulmoides*, one illustrative example comes from the anti-hypertensive literature: while individual iridoids (geniposidic acid) and lignans (PDG) each demonstrate hypotensive activity in vitro, the total iridoid fraction from *E. ulmoides* leaves failed to lower blood pressure in spontaneously hypertensive rats, and the combination of lignans with iridoids showed no synergistic effect [37]. This observation suggests that opposing pharmacological tendencies within the iridoid subclass—some members may activate counter-regulatory pathways—could neutralize the net hypotensive response. More broadly, studies on other botanicals have shown that structurally similar compounds can compete for the same molecular target, leading to antagonism; for example, 9-epi-artemisinin and artemisitene antagonize artemisinin activity in Artemisia annua by competing for heme-binding sites [88]. By analogy, certain iridoid isomers or glycosylation variants within *E. ulmoides* might exert mutually counteracting effects on shared targets such as ACE2 or eNOS, though this remains to be experimentally verified.

In summary, the “multi-component, multi-target, multi-pathway” paradigm that characterizes *E. ulmoides* action in metabolic syndrome is not merely a descriptive framework but is underpinned by measurable pharmacodynamic and pharmacokinetic interactions among its constituents. Future research should move beyond single-compound studies to systematically evaluate binary and higher-order combinations of iridoids, lignans, flavonoids, and polysaccharides using isobolographic analysis and synergy-directed fractionation [89]. Such approaches will be essential for optimizing standardized extracts and clarifying why, in many instances, the whole *E. ulmoides* extract proves more effective than any of its isolated parts.

## 4. The Interventional Effects of *Eucommia ulmoides* and Its Extracts on Metabolic Syndrome

**Figure 5:**
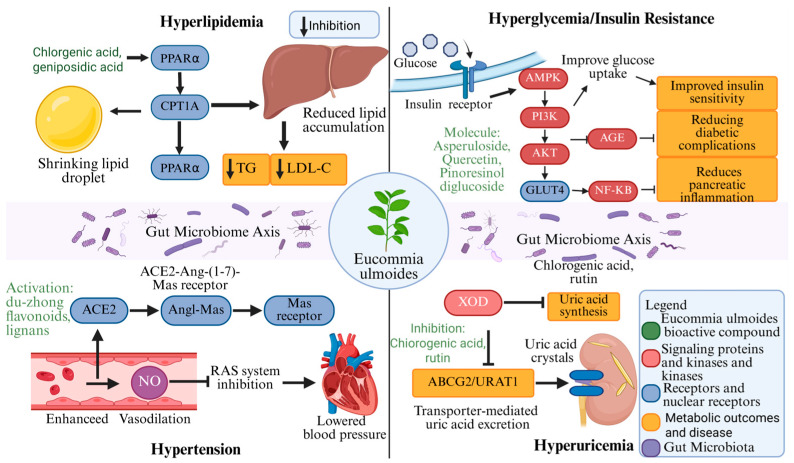
Integrated mechanistic diagram illustrating the multi-target interventions of *Eucommia ulmoides* bioactive constituents against the four core components of metabolic syndrome via gut microbiome modulation and key signaling pathways.

Eucommia exerts its therapeutic effects on metabolic syndrome (MetS) through a “multi-component, multi-target, multi-pathway” mechanism. This section provides a systematic review of Eucommia’s therapeutic effects on the four core features of metabolic syndrome—hyperlipidemia, hyperuricemia, hyperglycemia, and hypertension—from the following four perspectives: (1) pathophysiological background and current treatment limitations; (2) preclinical evidence from in vitro studies; (3) molecular mechanisms; and (4) the current status of clinical translation. To provide a comprehensive overview before detailed discussion, Figure 5 illustrates the integrated mechanistic framework through which bioactive constituents of *E. ulmoides* simultaneously target hyperlipidemia, hyperglycemia/insulin resistance, hypertension, and hyperuricemia, with the gut microbiome serving as a central modulatory axis.

### 4.1. Hyperlipidemia and Adiposity

#### 4.1.1. Pathophysiological Background and Therapeutic Challenges

Hyperlipidemia and obesity represent central components of MetS, characterized by dysregulated lipid metabolism, chronic low-grade inflammation, and insulin resistance, which collectively increase cardiovascular disease risk [90,91]. Hyperlipidemia, defined as LDL-C ≥ 130 mg/dL or non-HDL-C ≥ 160 mg/dL, drives atherosclerosis and premature myocardial infarction, with current management relying on lifestyle modification and statin-based pharmacotherapy [90]. Obesity (BMI ≥ 30 kg/m^2^), a chronic progressive disease, elevates the risk of dysglycemia, cardiovascular disorders, and cancer, shortening life expectancy by 5–20 years [91]. However, long-term adherence to lifestyle interventions remains poor, and polypharmacy increases the risk of adverse drug interactions [2,3].

#### 4.1.2. Preclinical Evidence for Lipid-Lowering Effects

A growing body of evidence indicates that extracts derived from different parts of *E. ulmoides*, particularly the leaves and bark, exert significant lipid-lowering effects in high-fat diet (HFD)-fed or chemically induced animal models.

Leaf extracts have been shown to improve hepatic lipid metabolism by suppressing fatty acid synthase and HMG-CoA reductase activities, enhancing fatty acid oxidation, and reducing hepatic lipid deposition in HFD-fed hamsters [92]. Aqueous leaf extracts administered to type 2 diabetic mice significantly lowered hepatic fatty acid synthase and HMG-CoA reductase activities while elevating lipoprotein lipase activity in skeletal muscle, thereby reducing plasma and hepatic lipid content [93]. Furthermore, *E. ulmoides* leaf extract ameliorates hepatic steatosis by enhancing lysosomal function, alleviating endoplasmic reticulum stress and oxidative stress, and promoting autophagic flux through downregulation of mTOR signaling [94]. In vitro studies using palmitate-induced HepG2 cells demonstrated that both aucubin and geniposide (25 μg/mL each) replicate these hypolipidemic effects by reducing ER stress and enhancing lysosomal activity [94].

Bark extracts also display protective effects in acute hepatic steatosis models by improving lipoprotein metabolism and reducing lipid accumulation [95], while chronic administration of bark extract in HFD-fed rats enhances metabolic function across multiple organs, including acceleration of hepatic β-oxidation and reduction in visceral adiposity [96].

Bioactive constituents in *E. ulmoides* leaf extract (EL), including asperuloside (ASP) and chlorogenic acid (CGA), have been reported to significantly reduce body weight, visceral fat mass, and serum levels of total cholesterol (TC), triglycerides (TG), and LDL-C, while increasing HDL-C levels in HFD-induced animal models [68,85,86,97,98] (Table 3). Among these constituents, asperuloside is considered one of the key active compounds and exhibits lipid-lowering effects comparable to those of the whole leaf extract [85,98].

#### 4.1.3. Molecular Mechanisms

Hepatic Lipid Metabolism Regulation. *E. ulmoides* leaves exert hypolipidemic effects primarily through activation of hepatic lipid metabolic pathways. Leaf extract markedly upregulates the hepatic expression of genes associated with fatty acid oxidation, such as PPARα and CPT1A, as well as key regulators of bile acid metabolism, including FXR, CYP7A1, and LXRα, while suppressing the expression of lipogenic genes such as SREBP-1c [85,86]. These coordinated changes promote the conversion of cholesterol into bile acids and thereby reduce hepatic lipid accumulation. Consistent with these molecular effects, histological analyses have shown a marked reduction in hepatic lipid deposition, with Oil Red O staining revealing significantly fewer intracellular lipid droplets, accompanied by decreased liver weight and liver index [86].

Recent network pharmacology studies further revealed that the active ingredients in *E. ulmoides* leaves regulating nonalcoholic fatty liver disease are mainly flavonoids and phenolics, which may play a role in lowering lipids by regulating PPARγ expression through inducing autophagy [99]. Additionally, *E. ulmoides* bark/leaf extracts significantly alleviate lipid metabolism disorders by affecting intestinal microbiota and microbiome–host interaction in HFD mice, demonstrating that both bark and leaf extracts can serve as alternative resources for anti-hyperlipidemic effects [100].

Oxidative Stress Mitigation. Asperuloside (ASP), one of the principal bioactive constituents of *Eucommia ulmoides* leaves, has been shown to markedly enhance the antioxidant capacity of hepatocytes through activation of the Nrf2/ARE signaling pathway, thereby upregulating downstream antioxidant genes, including HO-1, SOD2, and SIRT3 [86]. At the biochemical level, this activation is reflected by significant increases in hepatic superoxide dismutase (SOD) and catalase (CAT) activities, accompanied by a marked reduction in malondialdehyde (MDA) levels, indicating attenuation of oxidative stress [85]. In parallel, hepatic ATP levels are restored, mitochondrial function is improved, and dihydroethidium (DHE) staining reveals reduced reactive oxygen species (ROS) generation, further supporting the protective role of ASP against metabolic dysfunction-associated fatty liver disease (MAFLD) and obesity-related metabolic disturbances [86].

Gut Microbiota Modulation. *E. ulmoides* leaves improve lipid homeostasis through dose-dependent modulation of the gut microbiota in both composition and function. In animal models of high-fat diet-induced lipid metabolic disorder, *E. ulmoides* leaf extract ameliorates gut microbial dysbiosis. Medium to high doses (5.8–10.6 g·kg^−1^ or 256–512 mg·kg^−1^) significantly suppress pro-inflammatory taxa such as Proteobacteria and Erysipelotrichaceae, while increasing the abundance of beneficial and butyrate-producing bacteria, including Lactobacillus, Bacteroides, Akkermansia, Ruminococcaceae, and Prevotellaceae_NK3B31_group; by contrast, lower doses (3.2 g·kg^−1^ or 128 mg·kg^−1^) produce only modest regulatory trends [83]. These microbial shifts are accompanied by increases in alpha-diversity indices (Chao1, Ace, and Shannon), a beta-diversity profile that moves closer to that of normal controls, a significant reduction in the Firmicutes/Bacteroidetes ratio, and elevated fecal butyrate and total organic acid levels [68,86].

Mechanistic hypotheses for selective enrichment. The selective enrichment of Akkermansia muciniphila may involve three complementary mechanisms. First, dietary polyphenols (e.g., proanthocyanidins) serve as xenosiderophores: their catechol moieties chelate ferric iron, which *A. muciniphila* internalizes via ABC transporters, conferring a competitive growth advantage [101]. Second, polyphenols stimulate goblet cell differentiation and Muc2 secretion, expanding the mucin-rich niche that *A. muciniphila* preferentially colonizes [102]. Third, Bacteroides spp. produce N-acetylglucosamine during polysaccharide fermentation, which cross-feeds *A. muciniphila*, establishing a mutualistic trophic network [103]. For Bacteroides, enrichment is driven by their extensive polysaccharide utilization loci (PULs) encoding carbohydrate-active enzymes (CAZymes) that depolymerize *E. ulmoides* polysaccharide backbones (→4)-β-D-Glcp-(1→, →4)-α-D-GalpA-(1→) into fermentable oligosaccharides [104]. This cross-feeding is bidirectional: *A. muciniphila* mucin degradation releases oligosaccharides utilizable by Bacteroides, reinforcing their co-enrichment [105].

Notably, asperuloside has been specifically identified as a key modulator of gut microbiota. ASP administration changes the gut microbiota at the phylum level, decreasing Firmicutes and increasing Bacteroidetes, and at the genus level, increasing beneficial bacteria including Akkermansia, Parabacteroides, Roseburia, and Anaerostipes [106]. Akkermansia muciniphila, in particular, has been closely linked to the attenuation of insulin resistance and obesity, and its increase strengthens intestinal barrier function and reduces LPS-induced inflammation [106,107]. Fecal butyrate levels are negatively correlated with serum TC and TG concentrations (r < −0.5, *p* < 0.01) [68].

**Figure 6:**
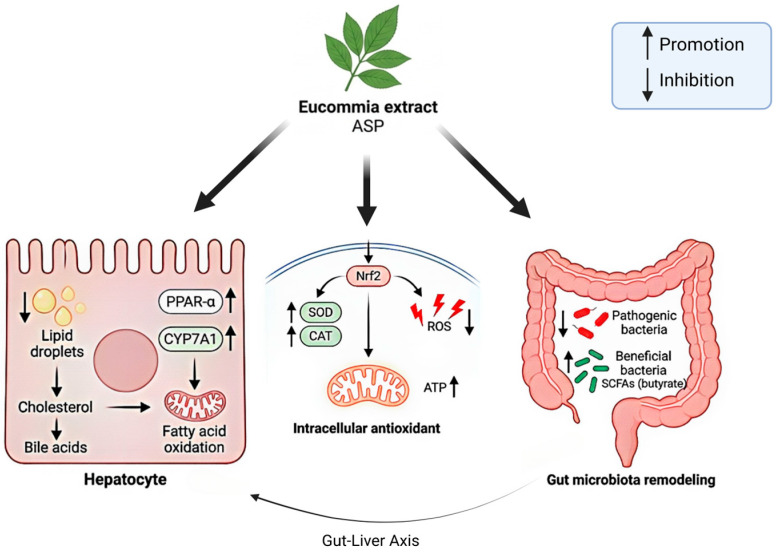
Mechanisms of *Eucommia ulmoides* and its extracts in hyperlipidemia regulation.

Fecal microbiota transplantation experiments further demonstrated that the remodeled microbial community can recapitulate the lipid-lowering phenotype even in the absence of direct drug exposure, primarily through activation of the bile acid synthetic axis (CYP7A1–FXR–LXRα) and GPR43-mediated fatty acid oxidation [68]. These findings suggest that *E. ulmoides* leaves alleviate hyperlipidemia via dose-dependent remodeling of the gut microbiota and subsequent regulation of the gut–liver axis. The proposed mechanism is summarized in Figure 6.

**Table 3:** Effects of active components in Eucommia on diseases caused by metabolic abnormalities in in vivo animal studies (↓ indicates downregulation; ↑ indicates upregulation).

	Related Disease	Cell Line/Animal Model	Treatment	Effects	References
Hyperlipidemia	Lipid metabolism disorders	HFD-induced C57BL/6J mice	EBE/ELE (320 mg/kg/day) for 12 weeks	↓ Serum TG, TC ↓ LDL-c↑ HDL-c ↓ Adipocyte hypertrophy	[68]
	obesity	HFD-fed Male Sprague–Dawley rats	Adding ELE and EGLP at 3 and 9%	↓ body weight and ↓ TAG and NEFA ↓ TNF-a ↓ ATP	[97]
		HFD-fed Sprague-Dawley rat	Adding 5% ELE and 0·03–0·3% ASP	↓ Insulin, lipid leve ↓ isocitrate dehydrogenase 3α	[98]
	Fatty liver	HFD-fed C57BL/6J male mice	ASP (50 mg/kg/day via oral gavage for 7 weeks)	↓ Total serum cholesterol ↓ Hepatic lipid ↑ mRNA: NPRA, HSL (lipolysis) ↓ Hepatic steatosis	[85]
		HFD-fed Wister rats	Extract of FEL (FELE) were 128 256 and 512 mg/kg/d	↓ TC, TG, LDL-C ↓ blood lipids ↓ lipocholic acid ↑ PPARα, CPT1A	[86]
Hyperuricemia	HUA	SD rats	(EU) was administered by intragastric gavage at 100, 200, or 400 mg/kg	↓ SUA ↑ OAT1/OAT3 mRNA ↓ URAT1/GLUT9 mRNA	[108]
		30 HUA volunteers	10 g of DZ daily for 1 week	↓ UA ↓ Cr ↓ BUN ↓ XOD	[109]
Hyperglycemia	Type 2 Diabetes	Diabetic SPF male C57BL/6 mice	10 mg/kg and 20 mg/kg, respectively	↓ liver index ↓ ALT, AST ↑ HDL-C, TG, TC and LDL-C ↑ Snail and α-SMA	[110]
		LX-2 cells	Additional 2.5 and 5 μM AU for 24 h.	↓ α-SMA and snail ↑ Ecadherin ↓ IL-1β and IL-18 ↓ p-IRE1α,TXNIP ↓ NLRP3,TGF-β1	[110]
	Hyperglycemia	The Kunming mice	Treated with 34 herbs for 4 weeks	↑ MDA	[111]
	Glucotoxicity	The C57BL/6 mice	EU, 200 mg/kg. The EU extract and AG were dissolved in distilled wate	↑ glyoxalase 1 ↑ Nrf2 ↓ receptor for AGE	[112]
Hypertension	Spontaneously hypertensive	Sprague–Dawley (SD) rats	EuL, 15, 30, 60 mg/kg	↓ BP of SHR ↓ SBP ↑ NO level ↓ Plasma RA ↓ Ang II	[113]
	Cardiac hypertrophy	Create an in vivo model of CM in SD rats	L-PDG, 2.5 μg/mL M-PDG, 5 μg/mL	↓ the cardiac mass index (HW/ BW, LVW/HW) ↓ ANP and BNP ↓ TNF-α, IL-6 and IL-1βat mRNA level	[84]
	High blood pressure and improved renal hemodynamics	Twenty-six DS rats	Treated with 500 mg/kg body weight of Tochu tea	↓ SBP in the DSHS rats ↑ RPF and GFR ↓ NADPH oxidase mRNA ↑ sodium excretion ↓ the expression of p47phox	[114]

Abbreviations: AG, aminoguanidine; AGE, advanced glycation end-products; ALT, alanine aminotransferase; α-SMA, alpha-smooth muscle actin; Ang II, angiotensin II; ANP, atrial natriuretic peptide; ASP, asperuloside; ATP, adenosine triphosphate; BNP, brain natriuretic peptide; BP, blood pressure; BUN, blood urea nitrogen; CM, cardiomyopathy; CPT1A, carnitine palmitoyltransferase 1A; Cr, creatinine; DS, Dahl salt-sensitive; DSHS, Dahl salt-sensitive hypertensive rats; DZ, Du-zhong (*Eucommia ulmoides*); EBE, Eucommia ulmoides bark extract; EGLP, Eucommia ulmoides gutta-percha leaf powder; ELE, Eucommia ulmoides leaf extract; EU, Eucommia ulmoides extract; EuL, Eucommia ulmoides leaf extract; FEL, fermented Eucommia ulmoides leaf; FELE, fermented Eucommia ulmoides leaf extract; GFR, glomerular filtration rate; GLUT9, glucose transporter 9; HDL-C, high-density lipoprotein cholesterol; HFD, high-fat diet; HSL, hormone-sensitive lipase; HUA, hyperuricemia; HW/BW, heart weight/body weight ratio; IL-1β, interleukin-1 beta; IL-6, interleukin-6; IL-18, interleukin-18; LDL-C, low-density lipoprotein cholesterol; LVW/HW, left ventricular weight/heart weight ratio; MDA, malondialdehyde; NADPH, nicotinamide adenine dinucleotide phosphate; NEFA, non-esterified fatty acids; NLRP3, NOD-like receptor thermal protein domain-associated protein 3; NO, nitric oxide; NPRA, natriuretic peptide receptor A; Nrf2, nuclear factor erythroid 2-related factor 2; OAT1/OAT3, organic anion transporter 1/3; p47phox, p47 phagocyte oxidase; p-IRE1α, phosphorylated inositol-requiring enzyme 1 alpha; PDG, pinoresinol diglucoside; PPARα, peroxisome proliferator-activated receptor alpha; RA, renin activity; RPF, renal plasma flow; SBP, systolic blood pressure; SD, Sprague-Dawley; SHR, spontaneously hypertensive rats; SPF, specific pathogen-free; SUA, serum uric acid; TAG, triacylglycerol; TC, total cholesterol; TG, triglycerides; TGF-β1, transforming growth factor beta 1; TNF-α, tumor necrosis factor-alpha; TXNIP, thioredoxin-interacting protein; UA, uric acid; URAT1, urate transporter 1; XOD, xanthine oxidase; AU, aucubin.

### 4.2. Hyperuricemia

#### 4.2.1. Pathophysiological Background and Therapeutic Challenges

Hyperuricemia (HUA) refers to a serum urate concentration exceeding its physiological solubility threshold, commonly defined as serum uric acid > 6.2 mg/dL in women and >7.0 mg/dL in men [115,116]. It arises from either excessive uric acid production or impaired urate excretion. As the terminal product of purine metabolism, uric acid is eliminated primarily through the kidneys (approximately 65–75%) and, to a lesser extent, through the intestine (25–35%) [116]. Hyperuricemia is closely associated with hypertension, MetS, type 2 diabetes, chronic kidney disease, and cardiovascular disease [117]. Current pharmacological options include xanthine oxidase inhibitors (allopurinol, febuxostat) and uricosuric agents (probenecid, benzbromarone), which are often limited by adverse effects such as hepatotoxicity and hypersensitivity reactions [115,116,117].

#### 4.2.2. Preclinical Evidence for Urate-Lowering Effects

Extracts derived from different parts of *Eucommia ulmoides*, particularly the leaves and bark, have been shown to ameliorate hyperuricemia-induced renal histopathology, lower serum creatinine (Cr) and blood urea nitrogen (BUN) levels, and thereby preserve renal function. In mice, daily gavage administration of an aqueous extract of *E. ulmoides* tea prepared from 10 g of leaves for 4 weeks markedly attenuated glomerular hypertrophy, narrowing of Bowman’s space, and vacuolar degeneration of renal tubules. These histopathological improvements were accompanied by a reduction in serum Cr from 53.6 to 36.2 μmol·L^−1^ and in BUN from 11.8 to 7.4 mmol·L^−1^ (*p* < 0.01) [109]. Similarly, in rats treated with an aqueous leaf extract at 200 mg·kg^−1^ for 8 weeks, glomerular adhesions were largely resolved, inflammatory infiltration was diminished, and serum Cr and BUN decreased by 28% and 31%, respectively, approaching values observed in normal controls [109,118].

#### 4.2.3. Molecular Mechanisms

Anti-inflammatory and Renal Protective Effects. *E. ulmoides* leaves mitigate hyperuricemia-associated inflammation by suppressing the expression of pro-inflammatory mediators. Their major active constituents, particularly flavonoids and iridoids, exert anti-inflammatory, immunomodulatory, and metabolic regulatory effects through several key signaling pathways, including the IL-17, TNF, and PI3K–AKT pathways, thereby indirectly alleviating the metabolic disturbances and tissue injury triggered by hyperuricemia [98]. Among these pathways, PI3K–AKT is critically involved in the regulation of cell survival, metabolic homeostasis, and inflammatory responses. By modulating PI3K–AKT signaling, *E. ulmoides* leaf constituents may improve renal urate transport capacity and enhance cellular adaptation to metabolic stress, ultimately attenuating urate-induced renal injury and systemic metabolic dysfunction [109].

Xanthine Oxidase Inhibition. *E. ulmoides* and its tissue-specific extracts exert urate-lowering effects through attenuation of oxidative stress and inhibition of uric acid biosynthesis. Gong et al. [118] reported that intervention with the aqueous extract of *E. ulmoides* leaf tea significantly reduced serum xanthine oxidase (XOD) activity in hyperuricemic mice, with the high-dose group showing levels close to those of the normal control group (*p* < 0.01). These findings indicate that *E. ulmoides* tea can suppress XOD activity, thereby inhibiting the sequential conversion of hypoxanthine to xanthine and xanthine to uric acid, and consequently lowering systemic uric acid levels [118]. Beyond direct enzymatic inhibition, the attenuation of oxidative stress represents an additional mechanistic pathway through which *E. ulmoides* extracts reduce uric acid biosynthesis. XOD is not only the terminal enzyme of purine catabolism but also a major endogenous source of reactive oxygen species (ROS), generating superoxide anion (O_2_^−^) and hydrogen peroxide (H_2_O_2_) as byproducts of urate production. Elevated ROS in turn further upregulates XOD activity, establishing a self-amplifying oxidative–hyperuricemic cycle. Active constituents of *E. ulmoides*, particularly chlorogenic acid and quercetin, have been shown to activate the Nrf2/HO-1 antioxidant signaling axis, thereby enhancing the expression of superoxide dismutase (SOD) and catalase (CAT) and restoring cellular redox homeostasis [119]. By simultaneously suppressing XOD-derived ROS generation and reinforcing endogenous antioxidant defenses, *E. ulmoides* extracts interrupt this vicious cycle at multiple nodes, resulting in coordinated reduction in both oxidative burden and uric acid synthesis [108].

Regulation of Renal Urate Transporters. Extracts from different parts of *Eucommia ulmoides* ameliorate hyperuricemia by promoting urate excretion through coordinated modulation of renal urate transporters. First, they enhance the expression of transporters involved in renal urate secretion. In mice, *E. ulmoides* bark extract (EU) administered at 160 or 320 mg·kg^−1^ significantly upregulated renal OAT1 and OAT3 expression; in rats, a higher dose of 400 mg·kg^−1^ was required to achieve comparable effects [108]. This increase in OAT1/OAT3 expression facilitates the transfer of urate from the circulation into the renal tubules, thereby lowering serum uric acid levels [108].

Second, *E. ulmoides* bark extract suppresses the expression of transporters associated with urate reabsorption, notably URAT1 and GLUT9, thus reducing tubular urate reuptake and promoting urinary excretion [108]. Recent research progress on multidimensional intervention strategies for hyperuricemia has highlighted that *E. ulmoides* cortex ethanol extract can significantly increase the mRNA expression of OAT1 and OAT3 in the kidneys of hyperuricemic rats, while simultaneously reducing the mRNA levels of GLUT9 and URAT1, proving its potential role in improving hyperuricemia through dual regulation of urate transport [120].

Third, several bioactive constituents of *E. ulmoides* leaves appear to interact directly with urate transport proteins. Molecular docking analyses have shown that flavonoids such as quercetin, kaempferol, and isoquercitrin, as well as iridoids including 8-epi-loganic acid and eucommiol C, exhibit binding affinities of ≤−5.9 kJ·mol^−1^ toward GLUT9, OAT1, and OAT3 [109]. At the in vivo level, administration of an aqueous leaf extract at 200 mg·kg^−1^ per day in rats—approximately equivalent to a human intake of 10 g leaves per day—significantly downregulated renal GLUT9 protein expression while upregulating OAT1 and OAT3, thereby inhibiting urate reabsorption, enhancing tubular secretion, and ultimately normalizing urate homeostasis [109].

**Figure 7:**
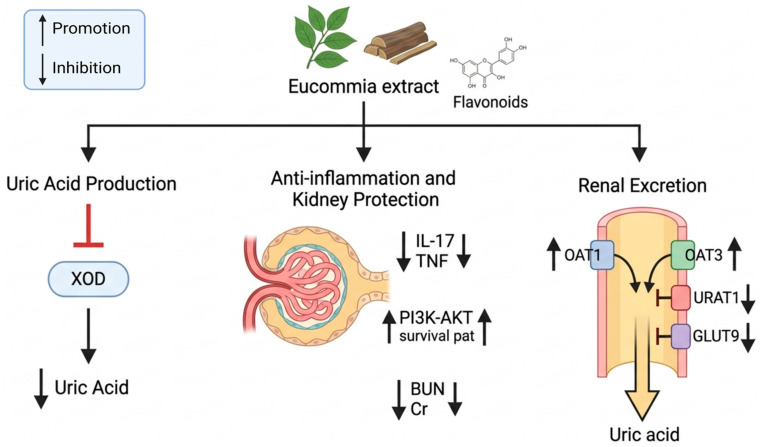
Mechanisms of *Eucommia ulmoides* and its extracts in hyperuricemia regulation.

Collectively, these findings indicate that extracts from both the bark and leaves of *E. ulmoides* alleviate hyperuricemia through a multi-target mechanism involving coordinated upregulation of OAT1/OAT3, downregulation of GLUT9/URAT1, XOD inhibition, and direct interaction with urate transporters, thereby restricting reabsorption and accelerating urate excretion. The proposed mechanism is illustrated in Figure 7.

### 4.3. Hyperglycemia

#### 4.3.1. Pathophysiological Background and Therapeutic Challenges

Hyperglycemia is defined as a fasting blood glucose level ≥ 100 mg/dL and encompasses both impaired fasting glucose (IFG, 100–125 mg/dL) and type 2 diabetes mellitus (≥126 mg/dL) [121]. As a central component of MetS, hyperglycemia acts synergistically with obesity, hypertension, and dyslipidemia to substantially increase the risk of chronic diseases. Sustained hyperglycemia promotes oxidative stress, chronic low-grade inflammation, and the accumulation of advanced glycation end products (AGEs), thereby damaging vascular endothelial cells and pancreatic β-cells [121,122]. Current management relies primarily on lifestyle intervention, with metformin as the first-line pharmacological agent, which exerts glucose-lowering effects mainly by improving insulin resistance and activating the AMPK pathway [123].

#### 4.3.2. Preclinical Evidence for Glucose-Lowering Effects

Accumulating evidence indicates that extracts from different parts of *Eucommia ulmoides*, particularly the leaves and bark, can lower hyperglycemia through coordinated multi-target mechanisms. These extracts enhance endogenous antioxidant enzyme activities, thereby attenuating oxidative stress and inflammatory injury, and activate the Nrf2–Glo1 pathway to facilitate methylglyoxal detoxification and inhibit the formation of AGEs [111,112,124,125].

Among the bioactive constituents of *E. ulmoides*, aucubin, an iridoid glycoside, has been identified as one of the principal compounds contributing to its antihyperglycemic activity [110]. Aucubin has been shown to enhance antioxidant enzyme activities in the liver and kidney, reduce lipid peroxidation, and thereby mitigate oxidative stress. In streptozotocin-induced hyperglycemic models, it also increases pancreatic β-cell number and insulin expression, resulting in significant glucose-lowering and pancreatic protective effects [126].

However, not all studies have observed a direct improvement in blood glucose. Bao et al. [110] reported that after 8 weeks of aucubin treatment, changes in blood glucose were not statistically significant, although glycated serum protein (GSP) levels were modestly reduced in diabetic mice receiving the high-dose regimen. These findings are consistent with those of Ma et al. [127] who likewise found that aucubin did not significantly alter blood glucose concentrations. Histomorphological analyses nevertheless indicate that aucubin exerts marked hepatoprotective effects, including attenuation of hepatic swelling, steatosis, and fibrosis [110].

#### 4.3.3. Molecular Mechanisms

Attenuation of Oxidative Stress and AGE Formation. *E. ulmoides* extracts ameliorate hyperglycemia by attenuating oxidative stress through multiple pathways. At 400 μg mL^−1^, the leaf polysaccharide fraction EUL-w1 increased superoxide dismutase (SOD) activity in insulin-resistant HepG2 cells from 104.25 to 137.92 U mg^−1^ (+33%) while reducing malondialdehyde (MDA) content from 4.02 to 2.67 nmol mg^−1^ (−41%), thereby markedly reversing high glucose-induced oxidative injury [125].

In addition, a 70% ethanol extract of *E. ulmoides* bark administered at 200 mg kg^−1^ for 6 weeks increased nuclear Nrf2 protein expression in the kidneys of diabetic mice by 1.5-fold and elevated glyoxalase 1 (Glo1) activity from 40 to 80 nmol min^−1^ mg^−1^ (+100%). These changes were accompanied by decreases of 45% and 50% in renal AGEs and methylglyoxal accumulation, respectively, indicating substantial relief of oxidative stress. Do et al. [112] further demonstrated that *E. ulmoides* bark extract ameliorates the detrimental effects of hyperglycemia through suppression of the AGEs/RAGE pathway. In streptozotocin-induced diabetic mice, treatment with bark extract (200 mg kg^−1^ for 6 weeks) reduced serum AGEs from 28 to 18 AU (−36%) and renal AGEs from 0.8 to 0.4 ng mg^−1^ protein (−50%). In vitro, 100 μg mL^−1^ of the extract inhibited BSA–glucose/fructose-derived AGE formation by 60% and reduced AGE–collagen cross-linking by 55%, demonstrating a marked ability to suppress both AGE generation and tissue deposition [112].

Recent studies have confirmed that *E. ulmoides* aqueous extract regulates oxidative stress through the Nrf2/HO-1 pathway and maintains calcium homeostasis in the kidney, intestine, and bone tissues, thereby ameliorating diabetic complications [128].

AMPK/PI3K/Akt Signaling and Hepatic Gluconeogenesis. Pathophysiologically, engagement of the receptor for AGEs (RAGE) by accumulated AGEs is a major driver of oxidative stress and inflammation in both host tissues and the gut microenvironment under diabetic conditions [129]. In parallel, hyperglycemia can suppress AMPK activity while exaggerating PI3K/AKT signaling, thereby amplifying oxidative stress in hepatic macrophages, promoting their pro-inflammatory M1 polarization, and exacerbating liver injury [130].

In an insulin-resistant HepG2 model, Gong et al. [125] found that EUL-w1 at 400 μg mL^−1^ increased AMPK mRNA expression from 0.36 to 0.47 (+29%), while restoring PI3K and Akt expression by 23% and 37%, respectively. These changes were accompanied by downregulation of the gluconeogenic enzymes PEPCK and G6Pase to near-normal levels and an increase in glucose consumption from 2.01 to 2.77 mmol L^−1^ (+38%), indicating significant suppression of hepatic gluconeogenesis and improvement of insulin resistance [125]. Recent research has further demonstrated that enzymatically extracted *E. ulmoides* polysaccharide (EUL-w1) exerts hypoglycemic activity on IR-HepG2 cells via the AMPK/PI3K/Akt signaling pathway, providing additional mechanistic insights into its antidiabetic potential [125].

Intestinal Carbohydrate Digestion and Glucose Absorption. *Eucommia ulmoides* and its tissue-specific extracts may improve hyperglycemia by suppressing intestinal carbohydrate digestion and glucose absorption. Evidence indicates that the 20% ethanol eluate of *E. ulmoides* leaves competitively inhibits α-glucosidase, with an IC50 value of 0.07 mg mL^−1^, showing potency comparable to that of acarbose. Moreover, *E. ulmoides* leaf extract at 1 mg mL^−1^ inhibited glucose transport in Caco-2 cells by 26% [124]. These findings suggest that *E. ulmoides* leaf extract exerts antihyperglycemic effects, at least in part, through inhibition of intestinal carbohydrate-hydrolyzing enzymes, including α-glucosidase, maltase, and sucrase, thereby limiting postprandial glucose release and absorption.

Hepatoprotective and Anti-fibrotic Effects. Mechanistically, aucubin appears to improve type 2 diabetes-associated liver injury by suppressing the NOX4/ROS pathway and interrupting the endoplasmic reticulum stress-driven IRE1α/TXNIP/NLRP3 inflammatory axis, thereby reducing ROS generation and pro-inflammatory mediator production. Through these actions, aucubin alleviates hepatic steatosis, inhibits hepatic stellate cell activation, and mitigates fibrosis [110]. Collectively, these findings suggest that aucubin, a representative constituent of *E. ulmoides* and its various tissues, may hold promise as a nutritional or therapeutic candidate for ameliorating type 2 diabetes-related hepatic injury and fibrosis.

**Figure 8:**
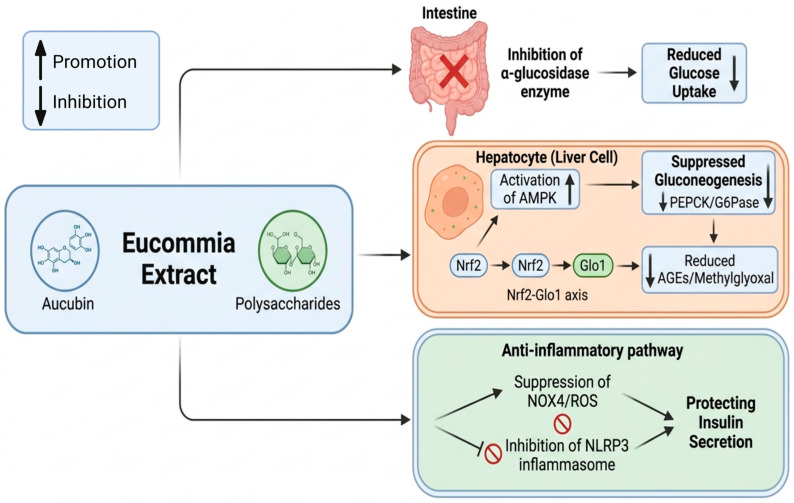
Mechanisms of *Eucommia ulmoides* and its extracts in hyperglycemia regulation.

The proposed mechanisms by which *E. ulmoides* and its extracts regulate hyperglycemia are summarized in Figure 8.

### 4.4. Hypertension

#### 4.4.1. Pathophysiological Background and Therapeutic Challenges

Hypertension is a chronic, progressive disorder and a major global public health challenge, affecting nearly 40% of adults worldwide [131]. The rising prevalence of hypertension has become one of the leading contributors to cardiovascular morbidity and mortality. Despite the availability of multiple antihypertensive agents, blood pressure remains inadequately controlled in an increasing proportion of patients [131]. As one of the hallmark components of MetS, hypertension is broadly classified into primary (essential) hypertension and secondary hypertension [132]. According to the World Health Organization, hypertension is responsible for approximately 9 million deaths annually, and it has been projected that by 2025, nearly 29.2% of the global adult population will be affected [133,134]. At present, clinical management relies predominantly on synthetic pharmacological agents; however, long-term use is often accompanied by adverse effects, underscoring the need for safer complementary strategies [135].

#### 4.4.2. Preclinical Evidence for Antihypertensive Effects

Owing to its potential antihypertensive activity, Eucommia folium (EF) has long been regarded as a promising natural candidate for antihypertensive drug development. As early as the 1950s, Russian investigators reported that EF exerted bidirectional regulatory effects on blood pressure, with a pronounced antihypertensive action considered superior to that of certain conventional chemical agents [136]. Since the 1970s, *Eucommia ulmoides* has been used in Sichuan, China, as an adjunctive therapy for hypertension and has also been widely consumed as a health-promoting food product [136]. In East Asian countries, including China, Korea, and Japan, EF has been extensively used for the prevention and treatment of cardiovascular diseases [137].

Current evidence suggests that *E. ulmoides* and its tissue-specific extracts improve hypertension through multiple complementary mechanisms. In addition to exerting direct vasorelaxant effects, they may also enhance nitric oxide (NO) production, attenuate oxidative stress, modulate inflammation-related signaling pathways, and regulate the renin–angiotensin system, thereby alleviating elevated blood pressure and its associated vascular abnormalities.

#### 4.4.3. Molecular Mechanisms

Nitric Oxide Production and Endothelial Function. Hosoo et al. [138] demonstrated that an ethanol extract of *E. ulmoides* leaves promotes NO production and improves endothelial function. In spontaneously hypertensive rats (SHRs), free access for 7 weeks to a diet containing 5% *E. ulmoides* leaf extract significantly increased plasma NO levels, enhanced endothelial nitric oxide synthase (eNOS) activity, and promoted vasodilation. These findings indicate that long-term administration of *E. ulmoides* leaf extract can markedly elevate circulating NO bioavailability in SHRs, improve endothelium-dependent relaxation of the aorta, and attenuate vascular hypertrophy [138].

Attenuation of Oxidative Stress. *Eucommia ulmoides* and its tissue-derived extracts may also exert antihypertensive effects through the attenuation of oxidative stress. In Dahl salt-sensitive hypertensive rats, 4 weeks of treatment with 1% saline containing either *E. ulmoides* tea extract (500 mg kg^−1^ day^−1^) or geniposidic acid (160 mg kg^−1^ day^−1^) significantly downregulated both the mRNA and protein expression of the NADPH oxidase subunits p47phox and gp22phox in the renal cortex, accompanied by a marked reduction in renal superoxide production. At the same time, endothelial nitric oxide synthase (eNOS) expression and NO production were increased, and renal plasma flow was improved. These changes were associated with substantial reductions in systolic blood pressure, from 196 mmHg to 158 mmHg in the *E. ulmoides* group and to 162 mmHg in the geniposidic acid group [114]. Collectively, these findings indicate that *E. ulmoides* tea and its major constituent geniposidic acid lower blood pressure by suppressing NADPH oxidase-driven oxidative stress, enhancing eNOS-derived NO bioavailability, and improving renal hemodynamics [114].

Recent reviews on the role of Chinese herbal medicine in regulating oxidative stress for hypertension have further confirmed that *E. ulmoides* exerts hypotensive effects through multiple pathways, including the RhoA/ROCK and NADPH oxidase/eNOS/NO/Ca^2+^ pathways, and can prevent and reverse renal damage caused by hypertension [139].

Anti-inflammatory and Cardioprotective Effects. Pinoresinol diglucoside (PDG), a bioactive constituent isolated from *Eucommia ulmoides*, has been reported to modulate hypertension, inflammation, and oxidative stress. Hypertension is a major risk factor driving the initiation and progression of myocardial hypertrophy. Persistent elevation of blood pressure can lead to adverse cardiovascular remodeling, compensatory left ventricular wall thickening, and myocardial fibrosis. In this context, PDG exerts cardioprotective effects by suppressing inflammatory signaling through the AKT/mTOR/NF-κB axis, thereby attenuating myocardial hypertrophy and fibrosis [84]. In rats subjected to abdominal aortic constriction (AAC), daily intraperitoneal administration of PDG (7.5 mg kg^−1^) for 3 weeks significantly reduced myocardial levels of phosphorylated AKT, phosphorylated mTOR, and nuclear NF-κB p65, while inhibiting IκB degradation. These molecular changes were accompanied by approximately 60%, 55%, and 50% reductions in myocardial TNF-α, IL-6, and IL-1β mRNA expression, respectively. Concomitantly, the left ventricular weight/body weight ratio decreased from 2.515 to 2.480 mg g^−1^, and the collagen volume fraction was reduced by nearly 40%, indicating marked attenuation of pressure overload-induced myocardial hypertrophy and interstitial fibrosis [84].

ACE2–Ang-(1–7)–Mas Axis Activation. *Eucommia ulmoides* and its tissue-derived extracts may also lower blood pressure by activating the ACE2–Ang-(1–7)–Mas axis and thereby promoting conversion of Ang II to Ang-(1–7). Ding et al. [102] found that oral administration of an aqueous extract of *E. ulmoides* male flowers (0.2 g mL^−1^, 1 mL kg^−1^) to spontaneously hypertensive rats (SHRs) for 7 weeks significantly upregulated ACE2 mRNA and protein expression in the renal cortex, reduced plasma Ang II by approximately 35%, and increased Ang-(1–7) levels by nearly twofold. Importantly, pretreatment with either the ACE2 inhibitor DX600 or the Ang-(1–7)–Mas receptor antagonist A–779 completely abolished the antihypertensive effect, confirming that this pathway is central to the blood-pressure-lowering action of the male flower extract [140]. Thus, *E. ulmoides* male flower extract lowers blood pressure in SHRs by upregulating ACE2, increasing Ang-(1–7) bioavailability, and activating Mas receptor signaling.

**Figure 9:**
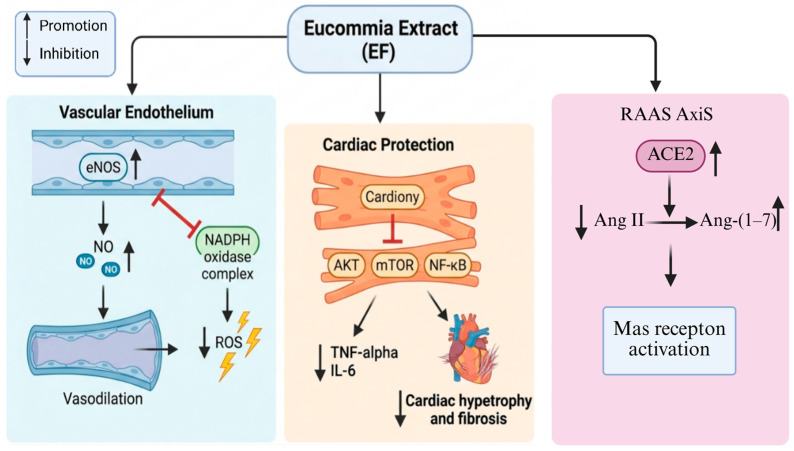
Mechanisms of *Eucommia ulmoides* and its extracts in hypertensionregulation.

Recent comprehensive reviews have confirmed that the antihypertensive activities of *E. ulmoides* may occur via activation of the ACE2-Ang-(1–7)-Mas signaling pathway, and that this mechanism may be present across different plant parts including male flowers, leaves, and bark [36,37]. The proposed mechanisms are illustrated in Figure 9.

### 4.5. Clinical Evidence and Translational Gap

While preclinical studies have provided robust mechanistic insights, clinical evidence for *E. ulmoides* in metabolic syndrome remains limited, with notable species-specific differences complicating translational efforts.

Regarding hypertension, Greenway et al. reported that a standardized bark extract (1 g t.i.d. for 2 weeks) reduced ambulatory BP by 7.5/3.9 mmHg in 30 adults with prehypertension, accompanied by β-adrenergic blocking activity. Xu et al. [141] confirmed these findings in a multicenter RCT of Quanduzhong capsules in 58 grade 1 hypertension patients, showing office BP reductions (−7.62/−4.66 mmHg) and decreased DBP variability versus placebo. Satonaka et al. [142] further demonstrated that Tochu leaf extract (85 mg GEA/day) lowered SBP in CKD patients (130.7 → 121.2 mmHg) with reduced oxidative stress and increased ANP. However, a critical species difference exists: GEA promotes ANP secretion via GLP-1R in rodents, but this axis is absent in humans [143]. Instead, bark-derived pinoresinol di-β-d-glucoside (PDG)—a cAMP-PDE inhibitor barely present in leaves—likely mediates human antihypertensive effects through β-adrenergic blockade [11,143].

Regarding obesity, clinical data are sparse and inconsistent. Nishibe et al. [143] noted no significant anti-obesity effects of leaf extract in Japanese subjects, whereas Zhou et al. reported reduced visceral fat (270.1 → 214.7 cm^2^), body weight, and BMI in 27 Chinese subjects with abdominal obesity after 8 weeks of leaf extract (1500 mg/day). This discrepancy may reflect dietary fiber-dependent gut microbiota modulation by asperuloside, suggesting rodent findings may not translate without appropriate dietary context.

Regarding other metabolic syndrome components, despite promising preclinical data for dyslipidemia, hyperglycemia, and hyperuricemia, robust clinical trials targeting these specific components of metabolic syndrome are currently lacking. The absence of large-scale RCTs evaluating *E. ulmoides* extracts for lipid-lowering, glycemic control, or urate-lowering effects in human subjects represents a significant limitation in the evidence base. While traditional use and small observational studies support the broader metabolic benefits of *E. ulmoides* tea consumption, these lack the methodological rigor required for evidence-based clinical recommendations.

#### Safety, Drug Interactions, and Contraindications

Human safety data. Long-term human data are limited. A 2-week RCT of standardized bark extract (1 g/day) reported mild headache, dizziness, and edema; serum creatinine (+0.02 mg/dL) and glucose (+3 mg/dL) changes were non-significant [11]. Rodent sub-chronic studies showed dose-dependent nephrotoxicity at 56 g/kg; lower-dose changes (11.2 g/kg) reversed within 5 weeks [10].

Drug interactions. (i) Antihypertensives: Additive hypotension risk via ACE2-Ang-(1-7)-Mas activation [140] and eNOS upregulation [138]; co-use with ACE inhibitors, ARBs, or β-blockers requires BP monitoring. (ii) Hypoglycemics: Aucubin and asperuloside activate AMPK/PI3K/Akt [125] and inhibit α-glucosidase [124]; may potentiate insulin/sulfonylurea/metformin effects. (iii) Anticoagulants: Theoretical bleeding risk; discontinue 2 weeks pre-surgery.

Contraindications. Absolute: Hypersensitivity; estrogen-dependent cancers (ERα-transactivating compounds detected [144]). Relative: Pregnancy/lactation (no safety data); renal impairment (nephrotoxicity risk at high doses [10]); hepatic impairment (genipin-related hepatotoxicity concern [34]); pediatric patients (no data).

## 5. Challenges and Future Perspectives

### 5.1. Research Limitations

Although *Eucommia ulmoides* shows considerable promise in improving metabolic syndrome-related parameters, the current evidence base remains subject to several important limitations. First, the vast majority of available data derive from animal models or in vitro experiments, whereas large-scale randomized controlled clinical studies confirming its efficacy and safety in humans are still lacking [145]. Second, the bioavailability, in vivo metabolic fate, and optimal delivery form of its active constituents, such as polysaccharides and flavonoids, remain insufficiently characterized, which hinders rational formulation optimization and product development [146]. Furthermore, doses in animal models are often empirically selected and extrapolated to humans without clear pharmacokinetic scaling. Based on the FDA dose conversion guidelines [147], the Human Equivalent Dose (HED) calculated from our reviewed preclinical data suggests that the estimated minimum effective dose of *Eucommia ulmoides* extracts for a 60 kg adult is approximately 48.6–145.8 mg/day (converted from 10 mg/kg in mice and 15 mg/kg in rats, respectively; detailed in Appendix A, Table A2). However, clinical trials often utilize much higher doses, such as 10 g of raw herb decoction daily [108]. This discrepancy highlights the critical need to optimize delivery forms. While traditional aqueous decoctions (teas) are widely used due to historical precedent and ease of preparation, they suffer from inconsistent constituent concentrations and poor stability. For rigorous clinical use, standardized extracts delivered via enteric-coated capsules or advanced microencapsulation systems are considered optimal. These modern delivery forms not only ensure a precise minimum effective dose but also enhance the low oral bioavailability of active phytochemicals (e.g., aucubin and chlorogenic acid) by protecting them from rapid enzymatic degradation in the gastrointestinal tract [148]. In addition, most existing studies have focused on single plant parts or isolated extracts, with comparatively limited investigation into the synergistic mechanisms among different tissues and multi-component combinations [149].

### 5.2. A Multidisciplinary Approach for Future Research

To bridge the translational gap between preclinical promise and clinical reality, future investigations on *E. ulmoides* in metabolic syndrome must adopt a multidisciplinary framework integrating pharmacology, microbiology, and nutritionology.

From the pharmacological perspective, the bioavailability and pharmacokinetic profiles of key bioactive constituents—particularly iridoid glycosides (e.g., geniposidic acid, aucubin) and lignan glycosides (e.g., pinoresinol diglucoside)—remain poorly characterized owing to their low intestinal permeability and extensive first-pass metabolism [150]. Systematic pharmacokinetic studies employing UPLC–Q-TOF–MS-based metabolomics are urgently needed to map the absorption, distribution, metabolism, and excretion (ADME) pathways of these compounds, as well as to identify their active metabolites generated through gut microbial biotransformation [14]. Furthermore, structure–activity relationship (SAR) studies should be conducted to guide the rational design of novel derivatives or prodrugs with enhanced oral bioavailability and target specificity.

From the microbiological perspective, the gut microbiota serves as a critical metabolic interface that governs the biotransformation, bioactivation, and therapeutic efficacy of *E. ulmoides* constituents. Emerging evidence demonstrates that Eucommia polysaccharides function as natural prebiotics that selectively enrich beneficial taxa such as Akkermansia, Lactobacillus, and butyrate-producing Ruminococcaceae [68], while concurrently enhancing the microbial hydrolysis of glycosides into pharmacologically active aglycones (e.g., genipin from geniposide) [13]. Integrated multi-omics approaches—including 16S rRNA sequencing, metagenomics, metabolomics, and transcriptomics—should be deployed to systematically characterize the host–microbiome co-metabolism of *E. ulmoides* bioactives and to identify microbial biomarkers predictive of therapeutic response [151]. Notably, recent studies on Cistanche polysaccharides have established a paradigm wherein polysaccharides enhance the oral bioavailability of co-administered small molecules (e.g., acteoside) by modulating gut microbiota diversity and short-chain fatty acid production. Analogous pharmacokinetic synergy between Eucommia polysaccharides and iridoid/lignan glycosides warrants rigorous investigation.

From the nutritional perspective, the integration of *E. ulmoides* into precision nutrition strategies offers a promising avenue for metabolic syndrome management. The chemical profile and bioactive content of *E. ulmoides* vary significantly across plant parts (bark, leaf, male flower, seed), harvest seasons, and processing methods [149], necessitating the development of chemotype-based quality control standards and personalized dietary supplement formulations. For instance, leaf extracts enriched in chlorogenic acid and asperuloside may be preferentially indicated for hyperglycemia and dyslipidemia, whereas bark extracts standardized for pinoresinol diglucoside may be more suitable for hypertension [140]. Moreover, the synergistic combination of *E. ulmoides* with other functional food ingredients—such as green tea polyphenols, citrus flavonoids, or dietary fibers—should be explored to enhance integrated metabolic regulatory effects through complementary mechanisms [149]. Nutritional intervention trials incorporating gut microbiota profiling and metabolomic phenotyping will be essential to validate the efficacy of such precision formulations in diverse human populations.

The convergence of these three disciplines—pharmacology, microbiology, and nutritionology—will enable the development of evidence-based, microbiota-targeted, and personalized *E. ulmoides* interventions for metabolic syndrome. This integrated approach aligns with the broader paradigm of systems medicine, wherein multi-omics data are leveraged to stratify patients, predict therapeutic outcomes, and optimize dosing regimens [118,119].

### 5.3. Potential for Translational Application

Future translational efforts should prioritize three directions. First, standardized *E. ulmoides* leaf extracts should be developed as dietary supplements or functional food ingredients for adjunctive metabolic management [149]. Second, synergistic formulations combining *E. ulmoides* with other natural products (e.g., green tea polyphenols, citrus flavonoids) warrant exploration to enhance integrated metabolic effects. Third, multi-omics technologies (metabolomics, transcriptomics, metagenomics) should be employed to systematically characterize the biosynthetic pathways of *E. ulmoides* bioactives and their host–microbiota metabolic networks in vivo [150,151,152,153]. Such approaches will enable chemotype-based quality control, biomarker identification, and precision intervention strategies tailored to different metabolic syndrome subtypes. Collectively, these efforts may facilitate the transition of *E. ulmoides* from a traditional medicinal resource toward an evidence-based precision health product.

## 6. Conclusions

The complex pathophysiology of metabolic syndrome necessitates therapeutic strategies capable of orchestrating multi-targeted interventions. *Eucommia ulmoides* Oliv., a traditional medicinal and edible plant, harbors a diverse array of bioactive constituents—including lignans, iridoids, flavonoids, and polysaccharides—that exert comprehensive modulatory effects on the core components of metabolic syndrome via a “multi-component, multi-target, multi-pathway” mechanism. In lipid metabolism, *E. ulmoides* promotes fatty acid oxidation through activation of the PPARα/CPT1A axis and ameliorates hyperlipidemia via the gut microbiota–liver axis. Blood pressure regulation is mediated by enhanced nitric oxide bioavailability and activation of the ACE2-Ang-(1–7)-Mas pathway, producing antihypertensive effects. In glucose homeostasis, it improves insulin resistance through the AMPK/PI3K/Akt signaling cascade and attenuates diabetic complications by inhibiting advanced glycation end-product formation. Additionally, uric acid metabolism is modulated through a dual mechanism—suppressing xanthine oxidase activity while regulating renal urate transporters to simultaneously inhibit production and promote excretion [68,86,109,110,125].

Despite these promising preclinical findings, significant limitations remain. Clinical translation is constrained by the paucity of high-quality randomized controlled trials validating efficacy and safety in humans. The bioavailability and pharmacokinetic profiles of active constituents are largely undefined, impeding formulation optimization [150]. Moreover, standardized quality control systems are still underdeveloped. Future research should prioritize rigorous clinical investigations, leverage multi-omics approaches to elucidate host–gut microbiome metabolic interactions, and develop precise interventions based on defined chemical profiles. With the integration of modern pharmaceutical technologies and systems biology, *E. ulmoides* holds potential to evolve from a traditional herbal remedy into an evidence-based natural intervention for metabolic syndrome, offering a novel strategy for disease prevention and management.

## Figures and Tables

**Table 1:** The Key Active Ingredients in Eucommia.

Ingredient Category	Representative Compounds	Structural Formula	Main Source
Iridoids	Genipinic acid	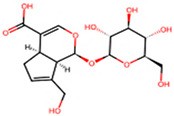	Leaf, Bark
	Aucubin	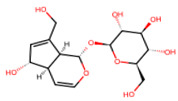	Leaf, Bark
Phenolic acids	Chlorogenic acid	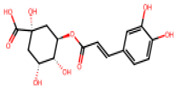	Leaf
Flavonoids	Quercetin	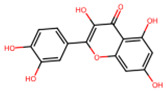	Leaf, Flower
	Rutin	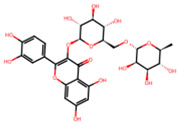	Leaf
Lignans	Pinoresinol diglucoside	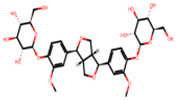	Bark
	4′-O-di-beta-D-glucopyranoside	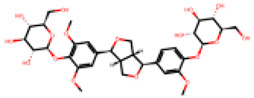	Bark
Polysaccharides	Eucommia polysaccharides		Bark, Leaf
Steroid	β-sitosterol	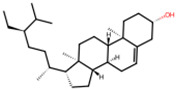	Bark, Leaf
Triterpenes	Oleanolic acid	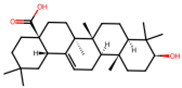	Leaf

**Table 2:** Pharmacokinetic parameters of key *E. ulmoides* constituents following oral administration in rats.

Tmax (h)	Dose (mg/kg)	Cmax (ng/mL)	Tmax (h)	T1/2 (h)	AUC0–t (ng·h/mL)	AUC0–∞ (ng·h/mL)	F (%)	Ref.
Geniposide	200 (extract)	1071.67 ± 598.46	1.54 ± 1.30	3.21 ± 2.31	4000.13 ± 1677.19	4119.45 ± 1649.61	~9.67	[75]
Genipin (aglycone)	200 (as geniposide)	76.44 ± 40.93	1.11 ± 0.40	3.42 ± 1.94	265.30 ± 103.43	356.72 ± 139.94	Very low	[75]
Aucubin	100	—	—	0.71 (β-phase)	—	—	~19.3	[76]
Pinoresinol diglucoside (PDG)	40	45.3 ± 37.5	0.50 ± 0.00	1.15 ± 0.75	113.0 ± 121.9	121.1 ± 128.9	~0.19	[56]

## Data Availability

The data presented in this study are available in the paper.

## Appendix A

**Table A1:** Extraction site and method.

Extraction Method	Extraction Part	References
Crushed separately before boiling in water for 4 h at a 1:10 ratio	Eucommia bark/leaf	[68]
Extract with 90 °C hot water for 1 h, filter, and concentrate the extract. Filter it and concentrate again, then Vacuum-dry to obtain a powder (yield: 18%).	Eucommia leaf	[97]
Extract with 90 °C hot water for 1 h, filter, and concentrate the extract. Filter it and concentrate again, then vacuum-dry to obtain a powder	Eucommia leaf	[98]
Extracted twice by reflux for one hour with 10 times the amount of water, filtered and combined the filtrate. The solvent was removed under vacuum	*Eucommia ulmoides* leaves	[86]
Perform two rounds of ethanol extraction, then evaporate and concentrate under vacuum.	The cortex of *E. ulmoides*	[108]
Soak in water for extraction twice, each time for 1.5 h. Filter the extract, then concentrate under reduced pressure and freeze-dry.	*Eucommia ulmoides* leaves	[118]
Extracted with 70% ethanol at room temperature overnight, filtered, evaporated, and then concentrated, followed by lyophilization using a freeze-dryer.	The bark of EU (*Eucommia ulmoides*)	[112]
Oaked in 90% ethanol (1:10, *w*/*v*) for 4 h at 80 °C, then filtered and concentrated with a vacuum rotary evaporator at 40 °C	*Eucommia ulmoides*	[111]
Macerationed in 60% EtOH at 70 °C for 2 h and extracted twice.	*Eucommia ulmoides* Oliv. bark	[113]

**Table A2:** Conversion of Animal Doses to Human Equivalent Doses (HED).

Disease Model	Animal Model	Administered Dose (Extract/Compound)	Conversion Coefficient	Calculated HED (mg/kg)	Estimated HED for a 60 kg Adult (mg/Day)	Ref.
Hyperuricemia	SD rats	100, 200, 400 mg/kg	0.162	16.2, 32.4, 64.8	972, 1944, 3888	[86]
Hyperglycemia	C57BL/6 mice	10, 20 mg/kg	0.081	0.81, 1.62	48.6, 97.2	[109]
Glucotoxicity	C57BL/6 mice	200 mg/kg (EU)	0.081	16.2	972	[112]
Hypertension	SD rats	15, 30, 60 mg/kg (EuL)	0.162	2.43, 4.86, 9.72	145.8, 291.6, 583.2	[113]

Note: HED is calculated based on the FDA guidance formula: HED (mg/kg) = Animal dose (mg/kg) × (Animal KmKm/Human KmKm). The conversion coefficient (KmKm ratio) is 0.162 for rats (Rat KmKm 6/Human KmKm 37) and 0.081 for mice (Mouse KmKm 3/Human KmKm 37), assuming a standard 60 kg human adult.

## Appendix B. Methods

### Appendix B.1. Literature Search Strategy

A systematic literature search was conducted using the following electronic databases: PubMed, Web of Science. The search was performed from inception to [insert date, e.g.,]. The following keywords and their combinations were used:

“*Eucommia ulmoides*”, “Du-zhong”, “iridoids”, “flavonoids”, “polysaccharides”, “lignans”, “phenolic acids”, “antioxidant”, “anti-inflammatory”, “neuroprotection”, “hepatoprotection”, “cardiovascular protection”, “antidiabetic”, “anti-obesity”, “gut microbiota”, “pharmacological activity”, “bioactive constituents”, “phytochemistry”

### Appendix B.2. Inclusion Criteria

Studies were included if they met the following criteria: Published in peer-reviewed journals in English or Chinese. Reporting pharmacological activities or bioactive constituents of *Eucommia ulmoides*. Including in vitro, in vivo, or clinical studies relevant to the topics covered in this review. Full text available for retrieval and assessment.

### Appendix B.3. Exclusion Criteria

Studies were excluded based on the following criteria: Duplicate publications or redundant reports. Studies with insufficient methodological detail or unreliable data. Conference abstracts, editorials, case reports, or non-peer-reviewed sources. Studies not directly related to the pharmacological or phytochemical properties of *E. ulmoides*.

### Appendix B.4. Study Selection and Data Extraction

Titles and abstracts of retrieved records were independently screened for relevance. Full texts of potentially eligible studies were subsequently assessed against the inclusion and exclusion criteria. Relevant data, including study design, biological activity, active constituents, proposed mechanisms, and key findings, were extracted and synthesized narratively to support the thematic structure of this review.
